# Preperitoneal Myelolipoma Occurring after Video-Assisted Thoracoscopic Surgery for Thymoma: A Case Report

**DOI:** 10.70352/scrj.cr.26-0192

**Published:** 2026-05-23

**Authors:** Chikara Nakagami, Sakiko Kumata, Chihiro Inoue, Hiroyuki Ogasawara, Tatsuaki Watanabe, Takayasu Ito, Jun-ichi Kobayashi, Ko Oriyama, Satoshi Kamata, Toru Kawakami, Ken Onodera, Yui Watanabe, Takaya Suzuki, Hirotsugu Notsuda, Hisashi Oishi, Yoshinori Okada

**Affiliations:** 1 Department of Thoracic Surgery, Institute of Development, Aging and Cancer, Tohoku University, Sendai, Miyagi, Japan; 2 Department of Anatomic Pathology, Tohoku University Graduate School of Medicine, Sendai, Miyagi, Japan; 3 Department of Surgery, Tohoku University Graduate School of Medicine, Sendai, Miyagi, Japan

**Keywords:** extra-adrenal myelolipoma, preperitoneal space, secondary surgery, previous surgical site, thymoma, video-assisted thoracoscopic surgery

## Abstract

**INTRODUCTION:**

Myelolipoma is a benign tumor composed of mature adipose tissue and hematopoietic elements that typically arises in the adrenal glands, and involvement of the anterior abdominal region is extremely uncommon. We report a rare case of myelolipoma arising in the subxiphoid preperitoneal region adjacent to the site where a sternum-lifting device had been placed during previous surgery.

**CASE PRESENTATION:**

A 62-year-old man had undergone extended thymectomy for thymoma associated with myasthenia gravis at the age of 39. The procedure was performed via bilateral video-assisted thoracoscopic surgery using a sternum-lifting technique. The tumor was classified as Masaoka stage III, and postoperative radiotherapy was administered. No recurrence was observed during 13 years of follow-up. Ten years after completion of follow-up, CT performed for an unrelated condition incidentally revealed a slowly enlarging 65 × 40-mm mass in the preperitoneal space, adjacent to the previous surgical site where a sternum-lifting device had been placed. MRI demonstrated a fat-containing component within the lesion, and the differential diagnosis included liposarcoma and teratoma. Complete surgical resection was therefore planned. A midline incision extending caudally from the xiphoid process was made, and en bloc resection of the tumor with the involved peritoneum was performed via laparotomy. Histopathological examination revealed mature adipose tissue admixed with hematopoietic elements, confirming the diagnosis of myelolipoma.

**CONCLUSIONS:**

This unique case of extra-adrenal myelolipoma highlights the importance of MRI-based qualitative assessment for tumors arising at unusual sites and underscores the need to include extra-adrenal myelolipoma in the differential diagnosis of fat-containing lesions occurring at previous surgical sites.

## Abbreviations

FDG
: fluorodeoxyglucose
FSI
: fat-suppression image
SUVmax
: maximum standardized uptake value
T1WI
: T1-weighted image
T2WI
: T2-weighted image

## INTRODUCTION

Myelolipoma is a rare benign tumor composed of mature adipose tissue and hematopoietic elements that most commonly arises in the adrenal glands. Extra-adrenal myelolipomas are uncommon, and their occurrence in the anterior abdominal region is exceedingly rare. Furthermore, the development of this tumor at a previous surgical site has rarely been described. We report a case of preperitoneal myelolipoma that developed adjacent to a previous surgical site following video-assisted thoracoscopic surgery for thymoma. The diagnostic challenges and the presumed etiology of this unusual case are discussed.

## CASE PRESENTATION

**Fig. 1:**
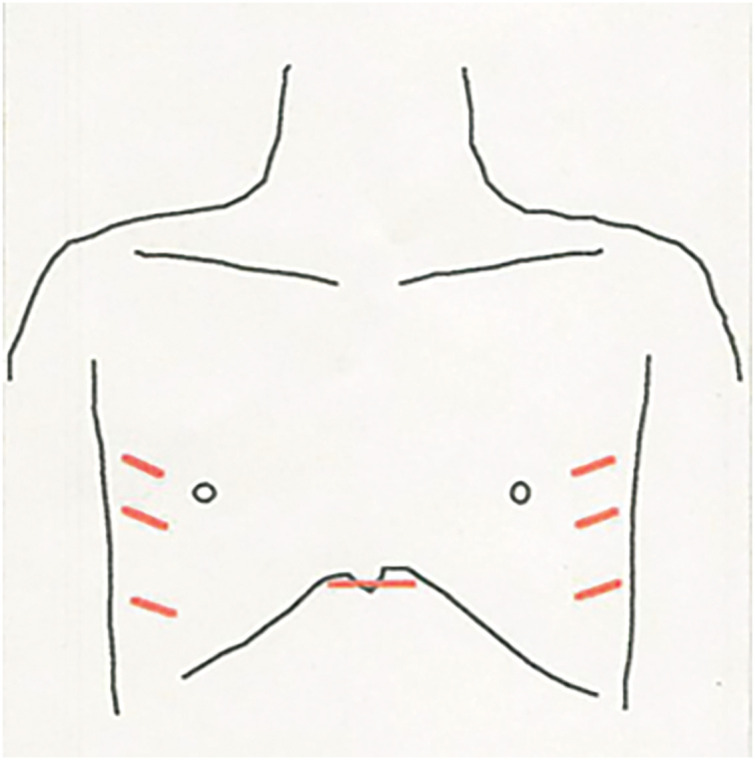
Previous surgical site incisions. During the initial surgery, thoracoscopic ports were inserted in the fourth, fifth, and seventh intercostal spaces bilaterally along the anterior axillary line, as well as in the subxiphoid region, as illustrated.

A 39-year-old man underwent surgery for thymoma associated with myasthenia gravis. The procedure consisted of bilateral video-assisted thoracoscopic extended thymectomy using a sternum-lifting technique. Thoracoscopic access ports were placed at 3 sites in each hemithorax, and an additional small subxiphoid incision was made for sternum lifting (**Fig. 1**). The resected thymoma was classified as Masaoka stage III because of invasion into the mediastinal pleura and pericardium. The surgical margin was pathologically positive, and postoperative radiotherapy to the anterior mediastinum was administered at a total dose of 50 Gy. Follow-up was discontinued 13 years after surgery, at which time no evidence of recurrence was observed.

**Fig. 2:**
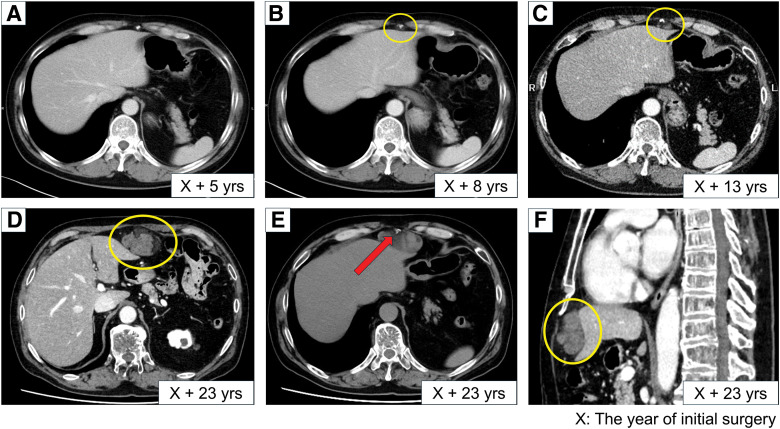
CT findings. (**A**) Five years after the initial surgery, no lesion was identified in the subxiphoid region. (**B**) Eight years after the initial surgery, a retrospective review revealed a very small area of increased fat attenuation in the subxiphoid region (yellow circle). (**C**) Thirteen years after the initial surgery, a retrospective review demonstrated a slight increase in the size of the subxiphoid lesion (yellow circle). (**D**) Twenty-three years after the initial surgery, the lesion had clearly enlarged (yellow circle). (**E**) Calcified components were observed within the lesion (red arrow). (**F**) A sagittal CT image obtained 23 years after the initial surgery, demonstrating the lesion in the subxiphoid region (yellow circle).

**Fig. 3:**
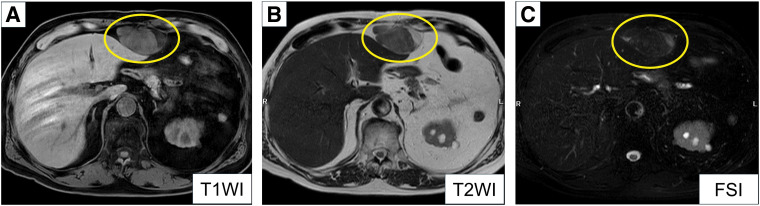
MRI findings. (**A**) T1WI demonstrated a lesion with mixed low- and high-signal intensities (yellow circle). (**B**) T2WI demonstrated a lesion with mixed low- and high-signal intensities (yellow circle). (**C**) On FSI, the signal intensity of the lesion decreased, indicating the presence of adipose tissue (yellow circle). FSI, fat-suppression image; T1WI, T1-weighted image; T2WI, T2-weighted image

At 62 years of age, 23 years after the initial surgery, the patient presented to a local clinic with lower extremity pain and was subsequently referred to our hospital, where he was diagnosed with lumbar spinal canal stenosis and spondylolisthesis. CT performed during this evaluation incidentally revealed a 65 × 40-mm mass in the preperitoneal space beneath the xiphoid process, near the previous surgical site. A retrospective review of prior CT images demonstrated subtle changes at the same site beginning 8 years after the initial surgery, and the lesion exhibited indolent growth over time. Compared with the most recent CT obtained 10 years earlier, the lesion had enlarged (**Fig. 2**). MRI demonstrated a lesion with mixed low- and high-signal intensities on both T1WIs and T2WIs. The signal intensity decreased on FSIs, suggesting the presence of adipose tissue (**Fig. 3**). Because the lesion was a progressively enlarging fat-containing tumor, liposarcoma was included in the differential diagnosis. Its midline location and the presence of focal calcification further raised the possibility of teratoma. Angiomyolipoma and extramedullary hematopoiesis were also considered in the differential diagnosis. However, angiomyolipoma was considered unlikely because it typically arises in the kidney or liver and because characteristic vascular features were absent. Extramedullary hematopoiesis was considered unlikely because the patient had no clinical findings suggestive of hematologic disease, such as anemia or splenomegaly. PET/CT demonstrated FDG uptake confined to the tumor, with a SUVmax of 3.0.

The patient’s medical history was otherwise unremarkable except for thymoma associated with myasthenia gravis. After extended thymectomy, he remained free of myasthenic symptoms while receiving prednisolone 10 mg every other day. He had a smoking history of 20 cigarettes per day for 18 years. His Eastern Cooperative Oncology Group performance status was 0.

Given the slow but progressive growth of the tumor, liposarcoma or teratoma could not be excluded based on MRI findings, and FDG uptake was observed on PET/CT. Therefore, surgical resection was planned. Surgery was performed under general anesthesia with the patient in the supine position. A 10-cm midline skin incision extending caudally from the xiphoid process was made, and the xiphoid process was subsequently excised. The tumor was surrounded by adipose tissue and was located in the preperitoneal space just beneath the xiphoid process, adjacent to the site where a sternum-lifting device had been placed during the prior surgery. The tumor was faintly palpable on manual examination. Foreign materials, such as surgical sutures, bone fragments, or scar tissue from the previous surgery, were not identified. Because the caudal aspect of the tumor was densely adherent to the peritoneum, en bloc resection of the tumor with the involved peritoneum was undertaken to achieve an adequate caudal margin. Consequently, the procedure was converted to laparotomy.

**Fig. 4:**
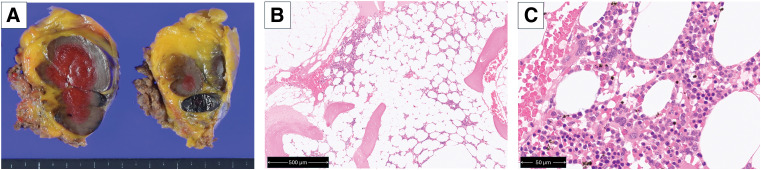
Pathological findings. (**A**) The lesion had a smooth brown outer surface and was firm on palpation. The center of the tumor appeared red because of suboptimal fixation. (**B**) Hematoxylin and eosin staining. A lower-power view showing a well-circumscribed mass composed of mature adipocytes and hematopoietic elements (scale bar: 500 μm). (**C**) A higher-power view demonstrated trilineage hematopoiesis, including megakaryocytes, granulocytes, and erythroblasts (scale bar: 50 μm).

Macroscopically, the excised tumor was firm and brownish on the surface. The cut surface was hemorrhagic and measured 6.5 cm at its greatest dimension. Microscopically, trilineage hematopoietic cells, including megakaryocytes, granulocytes, and erythroblasts, were observed within mature adipose tissue (**Fig. 4**). A small amount of trabecular bone formation was noted in the peripheral region of the tumor. The lesion was well circumscribed and clearly separated from the surrounding normal tissue. Hematopoietic cells were confined to the lesion and were not identified outside it. No atypical cells, including lipoblasts, were identified in the tumor. Based on the histopathological findings, the lesion was diagnosed as myelolipoma. Because the findings in the hematoxylin and eosin staining were typical and diagnostic, immunohistochemistry was not required. No thymoma components were identified. The tumor was well demarcated from the surrounding tissues, and no invasion of the peritoneum or rectus abdominis muscle was observed. Pathological examination confirmed complete resection.

## DISCUSSION

The reported incidence of adrenal myelolipoma at autopsy ranges from 0.08% to 0.4%, indicating that it is a relatively uncommon tumor.^1)^ Myelolipoma most commonly arises in the adrenal glands, and extra-adrenal myelolipomas account for approximately 10%–15% of all cases. Among extra-adrenal lesions, the presacral region is the most frequently reported site, followed by the liver, mediastinum, and retroperitoneum.^2,3)^

Myelolipoma arising in the anterior abdominal wall or preperitoneal space is extremely uncommon, and cases associated with prior surgical sites are also very limited. To our knowledge, only a single case involving the anterior abdominal wall at a previous surgical site has been reported in the literature by Pillay.^4)^ In that report, the lesion developed in the midline of the anterior abdominal wall over a decade after open mesh herniorrhaphy. Both the present case and the case reported by Pillay shared the feature that tumor formation became apparent about a decade after the initial surgery, suggesting a slowly progressive process over many years. In contrast, the lesion in the case reported by Pillay arose adjacent to nonabsorbable mesh, whereas no nonabsorbable material had been used at the tumor site in the present case.

Initially, recurrence of thymoma was suspected based on the CT findings. However, subsequent qualitative evaluation using MRI revealed that the tumor contained adipose tissue. At that stage, given that the lesion was a progressively enlarging midline mass containing fat and focal calcification, the main differential diagnoses included teratoma and liposarcoma. **Table 1** summarizes the imaging features of the major differential diagnoses in the present case.^1,3,5–15)^

**Table 1:** Imaging features of the major differential diagnoses in the present case

Diagnosis	CT findings	MRI findings	PET/CT findings	Findings relevant to the present case
Myelolipoma^1,3,5–7)^	Well-defined fat-containing mass with hematopoietic tissue; ± calcification/hemorrhage	High T1 and intermediate-to-high T2 signal; suppressed on fat-suppressed images	Mild FDG uptake; little to no uptake in the fatty component	Fat-containing lesion with mild FDG uptake
Well-differentiated liposarcoma^8,9)^	Lobulated heterogeneous mass with fatty areas	Predominantly high T1 signal with thick septa and nodular soft-tissue components; heterogeneous T2 signal (“triple sign”)	Mild to moderate FDG uptake	Progressive enlargement and nonfatty soft-tissue components
Mature teratoma ^8,10–12)^	Fat-containing lesion with calcification, soft-tissue, and occasionally cystic components	Mixed-intensity lesion with fat, fluid, soft tissue, and calcification	Usually low FDG uptake	Midline location and focal calcification
Immature teratoma^8,12,13)^	Heterogeneous lesion with fat, calcification, and solid components	Mixed signal intensity with irregular solid portions and less well-defined internal architecture	Moderate to high FDG uptake	Progressive enlargement, midline location, and focal calcification
Recurrent thymoma^12,14,15)^	Soft-tissue mass in the anterior mediastinum or prior operative field	Low to intermediate T1 signal and intermediate-to-high T2 signal; usually lacks macroscopic fat	Mild to moderate FDG uptake	History of thymoma resection and development in the prior operative field

FDG, fluorodeoxyglucose

The exact pathogenesis of extra-adrenal myelolipoma remains uncertain.^5,16,17)^ Several mechanisms have been proposed, and the most widely accepted theory is metaplastic transformation of mesenchymal cells induced by various stimuli, including inflammation, infection, and stress.^1,18–20)^ Another hypothesis suggests that microfractures of the vertebral bodies may allow hematopoietic tissue to extend into the paravertebral spaces. Such ectopic hematopoietic tissue may contain pluripotent stem cells capable of giving rise to extra-adrenal myelolipomas.^21)^

In the present case, the tumor developed adjacent to the previous surgical site in the subxiphoid region where a sternum-lifting device had been placed. It is therefore conceivable that surgical trauma and subsequent chronic inflammation triggered metaplastic transformation of local mesenchymal cells. Although reports of extra-adrenal myelolipomas arising at prior surgical sites are rare, it is noteworthy that the other anterior abdominal wall lesion reported by Pillay also developed at the site of a previous open mesh herniorrhaphy performed more than a decade earlier. Another possible explanation is that a small amount of bone marrow tissue was displaced during the sternal elevation procedure and subsequently implanted into the surrounding peritoneal tissues. However, because no bone-disrupting event was documented in the operative record, the likelihood of this hypothesis appears to be limited. Any association between tumor development and chronic mechanical irritation, surgical trauma, or ectopic implantation of bone marrow tissue remains speculative, and no direct evidence supporting these mechanisms was obtained in the present case.

In this case, although radiotherapy had been administered to the anterior mediastinum after the previous surgery, the subxiphoid region was outside the irradiation field. Therefore, any effect of prior radiotherapy was considered negligible.

For adrenal myelolipomas, conservative management is generally recommended for asymptomatic tumors smaller than 4 cm.^22,23)^ In contrast, surgical resection is considered for tumors measuring 6 cm or more, or for those demonstrating significant growth, because of the risks of spontaneous rupture or hemorrhage.^23,24)^ Owing to their rarity, no standardized treatment strategy has been established for extra-adrenal myelolipomas, and conservative management has been reported only for small, asymptomatic presacral lesions.^25)^ Surgical resection is recommended for symptomatic or progressively enlarging lesions, despite the absence of documented malignant transformation.

In the present case, the tumor demonstrated an indolent growth pattern, and based on imaging findings, the possibility of thymoma recurrence, teratoma, or malignant tumors such as liposarcoma could not be completely excluded. Therefore, surgical resection was ultimately considered an appropriate management strategy. This case highlights the importance of considering a broad differential diagnosis and carefully determining the indication for surgery when a lesion develops at a previous surgical site, rather than immediately concluding recurrence.

## CONCLUSIONS

We report a case of extra-adrenal myelolipoma arising in the subxiphoid preperitoneal region at the site where a sternum-lifting device had been placed during video-assisted thoracoscopic extended thymectomy performed 23 years earlier. This unique case highlights the importance of MRI-based qualitative assessment for tumors arising at unusual sites and underscores the need to include extra-adrenal myelolipoma in the differential diagnosis of fat-containing lesions occurring at previous surgical sites.

## DECLARATIONS

### Availability of data and materials

The datasets supporting the conclusions of this article are included within the article.
